# Antimicrobial resistance profiles in septic arthritis of native knee and shoulder joints from 2007 to 2024: a single center retrospective study

**DOI:** 10.3389/fmed.2025.1664798

**Published:** 2025-11-18

**Authors:** J. Straub, L. Willmann, D. Lee, J. Ortmayr, K. Staats, R. Windhager, C. Böhler

**Affiliations:** Department of Orthopedics and Trauma Surgery, Medical University of Vienna, Vienna, Austria

**Keywords:** septic arthritis, shoulder, knee, pathogens, susceptibility

## Abstract

**Introduction:**

Septic arthritis of large joints is associated with high morbidity and mortality, necessitating prompt empiric antibiotic therapy tailored to local and current pathogen patterns. The need for antimicrobial stewardship further highlights the importance of updated data on pathogens and resistance trends to guide effective empiric treatment.

**Methods:**

This study retrospectively investigated the distribution of causative pathogens and their antimicrobial resistance profiles in septic arthritis of native knee and shoulder joints from January 2007 to December 2024 at a tertiary hospital. A total of 326 patients who met the modified Newman Criteria and had confirmed culture results with antimicrobial susceptibility data were included. Cases involving prosthetic joints, treatment initiated at other facilities, or incomplete records were excluded. Cases were grouped into three six-year intervals to evaluate temporal trends in pathogen distribution and antimicrobial resistance.

**Results:**

*Staphylococcus aureus* was the predominant pathogen with 16 cases (51.61%) from 2007 to 2012, 25 (43.10%) from 2013 to 2018, and 23 (48.94%) from 2019 to 2024. Only six cases of *Methicillin-resistant S. aureus (MRSA)* were identified overall (4.41%). Other coagulase-negative staphylococci aside from *Staphylococcus epidermidis* decreased over time to 1 case (2.13%) from 2019 to 2024, while rates of gram-negative rods remained stable at about 10% over time. Enterococcal infections were rare, with only one case (0.74%), and there was only a single fungal infection (0.74%). A significant decline in resistance to Penicillin G was observed (*p* = 0.004), along with significant variations in clindamycin susceptibility, which showed a peak in resistance from 2013 to 2018 at 40.82% (*p* = 0.014). *Methicillin-sensitive S. aureus* predominated in younger adults 18–64 years (51.56%), whereas *Streptococcus* spp. were more frequent in patients over 65 years.

**Discussion:**

Our data support the empirical use of narrow spectrum beta-lactams for septic arthritis of the knee and shoulder. *MRSA* and gram-negative infections were rare, and high clindamycin resistance highlights the need to reconsider its routine use in patients with suspected penicillin allergy, emphasizing the need for antibiotic stewardship based on local pathogen and susceptibility distributions. Further, the age-related distribution of pathogens in our cohort, with *S. aureus* predominating in younger adults and *Streptococcus* spp. more frequent in older patients, is in line with previously reported trends.

## Introduction

Septic arthritis is defined as an acute infection of the joint caused by pathogens, which reach the joint either via haematogenous spread, direct inoculation, extension from adjacent infected tissue, or iatrogenic introduction (1). The incidence of septic arthritis varies globally, ranging from 1 to 35 cases per 100,000 individuals across different populations (2). Accurate diagnosis can be challenging since symptoms including joint pain, redness and swelling can also be attributed to several differential diagnoses such as osteoarthritis, gout and various rheumatological diseases, and joint aspirations are not always successful (1, 3). Due to its potential for systemic sepsis and mortality rate as high as 16.3% (2), acute septic arthritis is treated as an orthopedic emergency requiring rapid initiation of treatment to prevent irreversible joint destruction and minimize mortality (4).

Treatment for septic arthritis of large joints involves rapid surgical debridement either through arthroscopy or arthrotomy to remove infected tissue and relieve joint pressure and the use of intravenous antibiotics. While the surgical approach remains a topic of debate (5–7), the urgency and appropriateness of empirical antimicrobial therapy are universally acknowledged as pivotal to patient outcomes (4), as culture results may take days to show results, and PCR testing is not always readily available despite comparable diagnostic performance (8). Therefore, pathogen distributions and empirical antibiotic regimens play a critical role and are typically guided by local epidemiological data and resistance patterns (9). There is a vast array of bacteria capable of causing septic arthritis, with the most common pathogens in adults being *Staphylococcus aureus, Staphylococcus epidermidis* and *other coagulase negative Staphylococci, Streptococcus, Pseudomonas aeruginosa* and other gram-negative bacteria (2, 10).

The increasing prevalence of *methicillin-resistant S. aureus (MRSA)* and *vancomycin-resistant enterococci (VRE)* underscores the growing importance of evidence-based empirical antibiotic selection (11). Current recommendations advocate for initiating empiric therapy based on the most probable pathogens, with subsequent modification guided by culture and sensitivity results (1, 12). While several empiric treatment guidelines have been published (9, 12, 13), continuous surveillance and validation, particularly in relation to regional epidemiological and resistance trends, remain essential to maintaining clinical efficacy against resistant organisms (13).

Therefore, this retrospective single-center study aims to characterize the pathogens causing septic arthritis of native knee and shoulder joints in a tertiary hospital in central Europe between 2007 and 2024, along with their antimicrobial resistance profiles and temporal changes in resistance patterns.

## Methods

This 18 year retrospective study was conducted after approval by our local institution’s ethics committee (Nr. 1296/2019). Records were identified by ICD-10 codes and full-text searches for septic arthritis and related terms, yielding 734 cases admitted between January 2007 and December 2024.

Patients eligible for inclusion in this retrospective data analysis were adults over the age of 18 who had a diagnosis of septic arthritis of the knee or shoulder joint based on the modified Newman Criteria (14). Only patients with native joints, without prior prosthetic implantation, were considered. In addition, at least one positive culture from the infected tissue and/or blood, accompanied by an antimicrobial sensitivity analysis, was required for inclusion.

Patients were excluded from this study if they had undergone total knee or shoulder arthroplasty of the affected joint or if the diagnosis involved septic arthritis of a joint other than the knee or shoulder. Patients who had initiated treatment at a different medical facility were also excluded, as were those with incomplete medical records.

**Figure 1:**
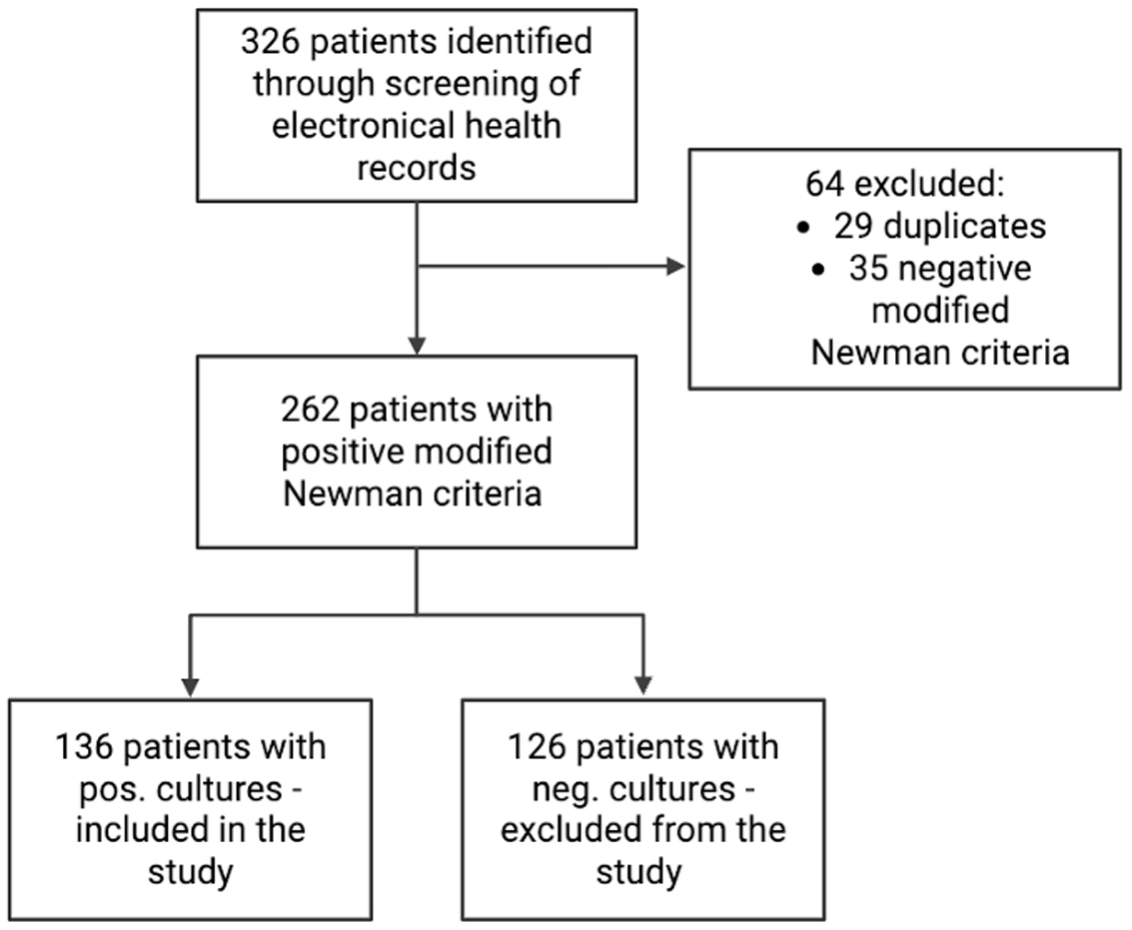
Flowchart of patient selection.

The patients` medical records, surgical report, bacterial culture and resistance profiles were then manually collected according to our inclusion and exclusion criteria. A total of 326 patients were included in the final analysis (cf. Figure 1). Furthermore, the patients’ past medical histories were reviewed to identify potential causes of septic arthritis. The analyzed causes included infections occurring up to 37 days following arthroscopic surgery (15), up to 65 days after intra-articular injection (16), haematogenous spread, or unknown etiology.

### Case grouping

This study was divided into the following three 6-year time periods: January 2007 to December 2012 (2007–12), January 2013 to December 2018 (2013–18) and January 2019 to December 2024 (2019–24). The distribution of the different pathogens across various time periods, age groups, and infection types, as well as their resistance to antimicrobial agents were analyzed.

Additionally, data were collected on the virulence of identified pathogens, distinguishing between high- and low-virulent organisms (17). The proportion of *MRSA* was recorded. Temporal trends were analyzed to assess potential dynamics over the three defined time intervals.

### Microbiological cultures

Samples of synovial fluid were cultured on a set of different media including Columbia Agar III with 5% Sheep Blood (BD, Heidelberg, Germany), chocolate agar supplemented with IsoVitaleX™ and bacitracin (BD), and MacConkey agar Nr. 3 (Thermo Fisher Scientific Oxoid Ltd., Basingstoke, United Kingdom) were incubated at 35–37 °C in a CO₂-enriched atmosphere for up to 2 weeks.

For culturing anaerobic pathogens, Brucella Blood Agar with Hemin and Vitamin K1 (BD, Heidelberg, Germany) and Schaedler Kanamycin-Vancomycin agar with 5% Sheep Blood (BD, Heidelberg, Germany) were used. Additionally, samples were inoculated into brain heart infusion supplemented with 0.1% agar (Oxoid, Wesel, Germany) and incubated for up to 2 weeks.

### Statistical analysis

Descriptive statistics were used to display baseline characteristics, pathogen distributions and drug resistance. Absolute values and percentages were used to describe categorial variables, and median values and inter quartile ranges were used for metric baseline characteristics. A chi-squared test was employed to compare categorical variables and Fisher’s exact test in case of less than five observations. A Kruskal-Wallis test was used to compare three groups of metric variables. Kaplan–Meier survival curves were used to illustrate and compare survival between gram-positive and gram-negative cases, with significant differences assessed using the log-rank test. Statistical analysis was conducted using R Version 4.4.1 at a significance level of 0.05 (18).

## Results

### Demographic results

A total of 136 patients were included in the final analysis after patient selection as depicted in Figure 1. 31 cases (22.79%) occurred between 2007 and 2012, 58 cases (42.64%) between 2013 and 2018, and 47 cases (34.55%) between 2019 and 2024. The baseline characteristics and demographics of the patients are summarized in Tables 1, 2.

**Table 1:** Baseline characteristics, values are either in absolute numbers and percentages or median and interquartile range.

Baseline characteristics	Overall (*n* = 136)	2007–2012 (*n* = 31)	2013–2018 (*n* = 58)	2019–2024 (*n* = 47)	*p*-value
Age (Median [IQR])	66.10 [48.72; 79.75]	65.30 [38.76; 72.91]	69.65 [51.61; 78.64]	63.40 [48.13; 80.94]	0.36
Gender					
Male	85 (62.50%)	23 (74.19%)	29 (50.00%)	33 (70.21%)	**0.03**
Female	51 (37.50%)	8 (25.81%)	29 (50.00%)	14 (29.79%)	
BMI (Body Mass Index, median [IQR])	25.37 [22.93; 28.65]	25.71 [24.21; 27.76]	25.62 [22.39; 29.20]	24.79 [22.98; 28.04]	0.21
Joint					
Knee	99 (72.79%)	20 (64.52%)	42 (72.41%)	37 (78.72%)	0.38
Shoulder	37 (27.21%)	11 (35.48%)	16 (27.59%)	10 (21.28%)	
Duration of symptoms (days), (median [IQR])	5 [2.00; 9.25]	7 [2.50; 21.00]	4 [3.00; 8.00]	5 [2.00; 7.50]	0.61

Values are given in absolute numbers (*n*) and percentages (%) for discrete data, and as median and inter quartile range (IQR) for metric data.

**Table 2:** Demographic characteristics and comorbidities.

Comorbidities	Overall (*n* = 136)	2007–2012 (*n* = 31)	2013–2018 (*n* = 58)	2019–2024 (*n* = 47)	*p*-value
Insulin dependent diabetes	10 (7.35%)	2 (6.45%)	4 (6.90%)	4 (8.51%)	0.99
Non-insulin dependent diabetes	29 (21.32%)	7 (22.58%)	12 (20.69%)	10 (21.28%)	
Chronic kidney disease	20 (14.71%)	2 (6.45%)	8 (13.79%)	10 (21.28%)	0.19
HIV	3 (2.21%)	0 (0.00%)	1 (1.72%)	2 (4.26%)	0.43
Hepatitis C	8 (5.88%)	1 (3.23%)	4 (6.90%)	3 (6.38%)	0.77
Gout	9 (6.98%)	6 (19.35%)	2 (3.92%)	1 (2.13%)	**0.01**
Ethanol abuse	5 (3.68%)	2 (6.45%)	3 (5.17%)	0 (0.00%)	0.24
Intravenous drug use	11 (8.09%)	1 (3.23%)	5 (8.62%)	5 (10.64%)	0.49
Rheumatoid arthritis	2 (1.47%)	1 (3.23%)	1 (1.72%)	0 (0.00%)	0.50
Immunosuppressive therapy	12 (8.82%)	2 (6.45%)	5 (8.62%)	5 (10.64%)	0.81
CCI (Median [IQR])	3 [1.00; 5.00]	2 [0.50; 4.00]	3 [2.00; 5.00]	4 [1.00; 6.00]	0.39
Gächter score (Median [IQR])	2 [2.00; 2.25]	2 [2.00; 2.00]	2 [2.00; 2.50]	2 [2.00; 3.00]	0.30

Values are given in absolute numbers (*n*) and percentages (%) for discrete data, and as median and inter quartile range (IQR) for metric data. CCI denotes the Charlson Comorbidity Index.

### Pathogen results

The pathogens isolated from the included patients were summarized into 13 different groups, with gram-positive bacteria clearly being the dominant cause of septic arthritis. A total of 121 (88.97%) gram-positive pathogens were identified over the studies period, versus 14 (11.03%) infections with gram-negative bacteria (Table 3). Over the studied period, the rate of gram-positive infections stayed almost constant with 90.32% (2007–2012), 87.93% (2013–2018) and 89.36% (2019–2024). The main bacteria identified was *methicillin sensitive S. aureus (MSSA)* (51.47%), followed by *Streptococcus* spp. (22.06%). The remaining pathogens *MRSA, Staphylococcus epidermidis, other coagulase negative Staphylococcus, Cutibacterium acnes, Enterococcus faecalis, Pseudomonas aeruginosa, Escherichia coli, Citrobacter koseri, Haemophilus influenzae* and *Candida albicans* were all sparsely available with a total isolation number of 10 or less (cf. Table 3).

**Table 3:** List of pathogens identified via positive cultures.

Pathogens	Total	2007–12	2013–18	2019–24
	(*n* = 136)	(*n* = 31)	(*n* = 58)	(*n* = 47)
	No. (%)	No. (%)	No. (%)	No. (%)
Gram-positive	**121 (88.97)**	**28 (90.32)**	**51 (87.93)**	**42 (89.36)**
*S. aureus (MSSA)*	64 (47.06)	16 (51.61)	25 (43.10)	23 (48.94)
*MRSA*	6 (4.41)	0 (0.00)	5 (8.62)	1 (2.13)
*S. epidermidis*	10 (7.35)	3 (9.68)	6 (10.34)	1 (2.13)
*Other coagulase neg. Staphylococci*	8 (5.88)	3 (9.68)	4 (6.90)	1 (2.13)
*Streptococcus* spp.	30 (22.06)	6 (19.35)	9 (15.52)	15 (31.91)
*C. acnes*	2 (1.47)	0 (0.00)	1 (1.72)	1 (2.13)
*E. faecalis*	1 (0.74)	0 (0.00)	1 (1.72)	0 (0.00)
Gram-negative	**14 (10.29)**	**3 (9.68)**	**6 (10.34)**	**5 (10.64)**
*P. aeruginosa*	7 (5.15)	1 (3.22)	3 (5.17)	3 (6.38)
*E. coli*	4 (2.94)	0 (0.00)	3 (5.17)	1 (2.13)
*C. koseri*	1 (0.74)	0 (0.00)	0 (0.00)	1 (2.13)
*H. influenzae*	1 (0.74)	1 (3.22)	0 (0.00)	0 (0.00)
*N. gonorrhoeae*	1 (0.74)	1 (3.22)	0 (0.00)	0 (0.00)
Fungi	**1 (0.74)**	**0 (0.00)**	**1 (1.72)**	**0 (0.00)**
*C. albicans*	1 (0.74)	0 (0.00)	1 (1.72)	0 (0.00)

Values in absolute numbers (*n*) and percentages (%).

In 39 cases (28.68%), intra articular injections were reported shortly before symptom onset, 7 cases (5.15%) occurred as postoperative complications, 7 cases (5.15%) were associated with injections related to intravenous drug use, and in 9 cases (6.62%) the infection spread from other infectious sites in the body.

Among patients with a history of intra articular injections as most probable cause for their septic arthritis (*n* = 39), the most frequently identified pathogen was *Staphylococcus aureus* (*n* = 18, 46.15%), followed by *Streptococcus* spp. (*n* = 10, 25.64%), *S. epidermidis* (*n* = 5, 12.82%), *other coagulase-negative staphylococci* (*n* = 3, 7.69%), *MRSA* (*n* = 3, 7.69%), and *P. aeruginosa* (*n* = 1, 2.56%). There was no significant difference in pathogen distribution between patients with intra-articular injections and patients with septic arthritis from other causes (*p* = 0.55).

Age-stratified results indicated a decline in *S. aureus* infections among individuals over 65, accompanied by an increase in *Streptococcus* spp. infections in this age group (cf. Table 4).

**Table 4:** Pathogens identified from positive cultures across different age groups.

Pathogens	Total	18–64 years	65–79 years	> 80 years
	(*n* = 136)	(*n* = 64)	(*n* = 39)	(*n* = 33)
	No. (%)	No. (%)	No. (%)	No. (%)
Gram-positive	**121 (88.97)**	**56 (87.50)**	**37 (94.87)**	**28 (84.84)**
*S. aureus (MSSA)*	64 (47.06)	33 (51.56)	17 (43.59)	14 (42.42)
*MRSA*	6 (4.41)	2 (3.13)	3 (7.69)	1 (3.03)
*S. epidermidis*	10 (7.35)	6 (9.38)	3 (7.69)	1 (3.03)
*Other coagulase neg. Staphylococci*	8 (5.88)	5 (7.81)	2 (5.13)	1 (3.03)
*Streptococcus* spp.	30 (22.06)	8 (12.50)	12 (32.43)	10 (30.30)
*C. acnes*	2 (1.47)	2 (3.13)	0 (0.00)	0 (0.00)
*E. faecalis*	1 (0.74)	0 (0.00)	0 (0.00)	1 (3.03)
Gram-negative	**14 (10.30)**	**7 (10.94)**	**2 (5.13)**	**5 (15.15)**
*P. aeruginosa*	7 (5.15)	2 (3.13)	2 (5.13)	3 (9.09)
*E. coli*	4 (2.94)	2 (3.13)	0 (0.00)	2 (6.06)
*C. koseri*	1 (0.74)	1 (1.56)	0 (0.00)	0 (0.00)
*H. influenzae*	1 (0.74)	1 (1.56)	0 (0.00)	0 (0.00)
*N. gonorrhoeae*	1 (0.74)	1 (1.56)	0 (0.00)	0 (0.00)
Fungi	**1 (0.74)**	**1 (1.72)**	**0 (0.00)**	**0 (0.00)**
*C. albicans*	1 (0.74)	1 (1.72)	0 (0.00)	0 (0.00)

Values in absolute numbers (*n*) and percentages (%).

**Figure 2:**
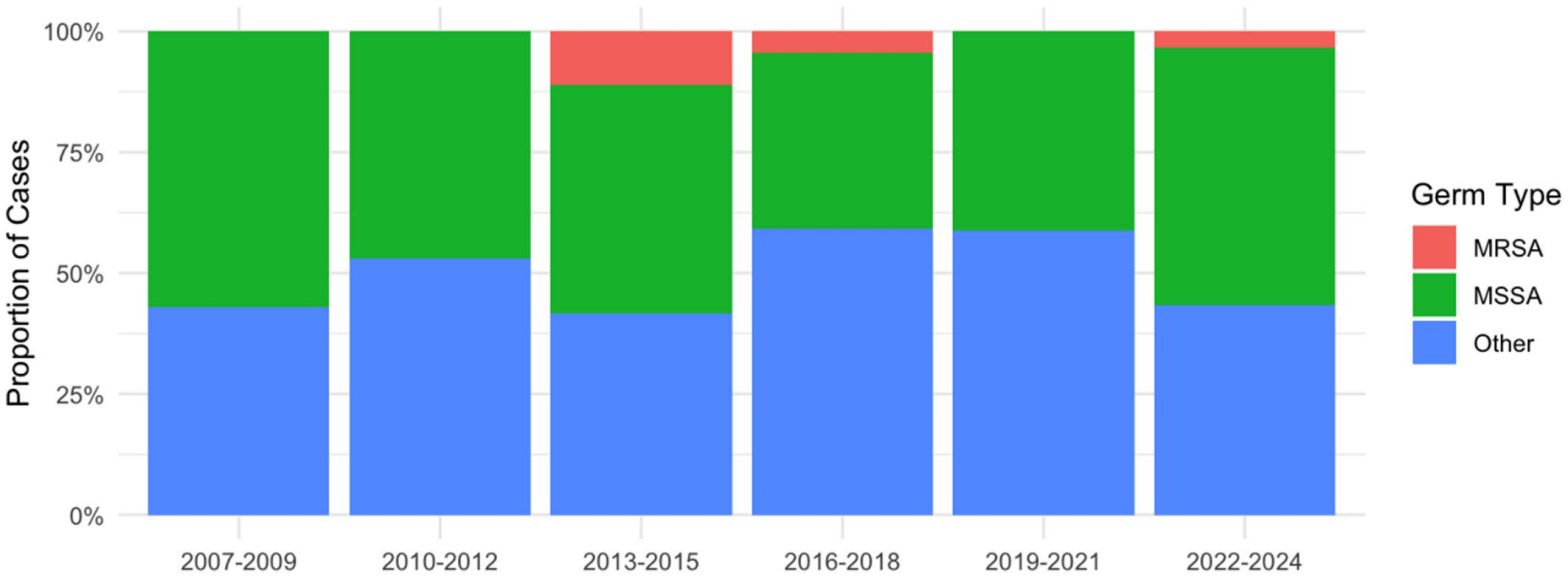
Relative distribution of MRSA, MSSA and other pathogens in 3-year intervals.

For *S. aureus*, 6 out of 70 cases (8.6%) were considered *MRSA*, each case showing a resistance against oxacillin, erythromycin and clindamycin (Figure 2). Two of the cases occurred in 2014, two in 2015, one in 2018 and one in 2023, and all six were susceptible to vancomycin, linezolid, teicoplanin, and tigecycline and one resistance to doxycycline was seen in 2018. As for *MSSA* infections, all 21 isolates tested for cefazolin between 2019 and 2024 were susceptible, however, cefazolin was not tested directly in earlier periods. Clindamycin resistance was observed in 3 of 16 isolates (18.75%) from 2007 to 2012, 5 of 25 isolates (20.00%) from 2013 to 2018, and 4 of 23 isolates (17.39%) from 2019 to 2024. Notably, all isolates resistant to clindamycin also exhibited resistance to erythromycin.

For *Streptococcus* spp., no penicillin resistance was detected in any of the study periods. Clindamycin resistance was absent from 2007–2012, but emerged in 2013–2018 with 55.56% (5 of 9) resistant isolates, and declined in 2019–2024 with 20.00% (3 of 15). Erythromycin resistance followed a similar pattern, with no resistant isolates in 2007–2012, but 50.00% (2 of 4) in 2013–2018 and 14.29% (1 of 7) in 2019–2024.

Among the seven *P. aeruginosa* isolates, all were susceptible to the tested antibiotics except for a single isolate from 2022, which showed resistance to meropenem, cefepime, imipenem, and aztreonam, while remaining susceptible only to piperacillin–tazobactam and tobramycin.

**Figure 3:**
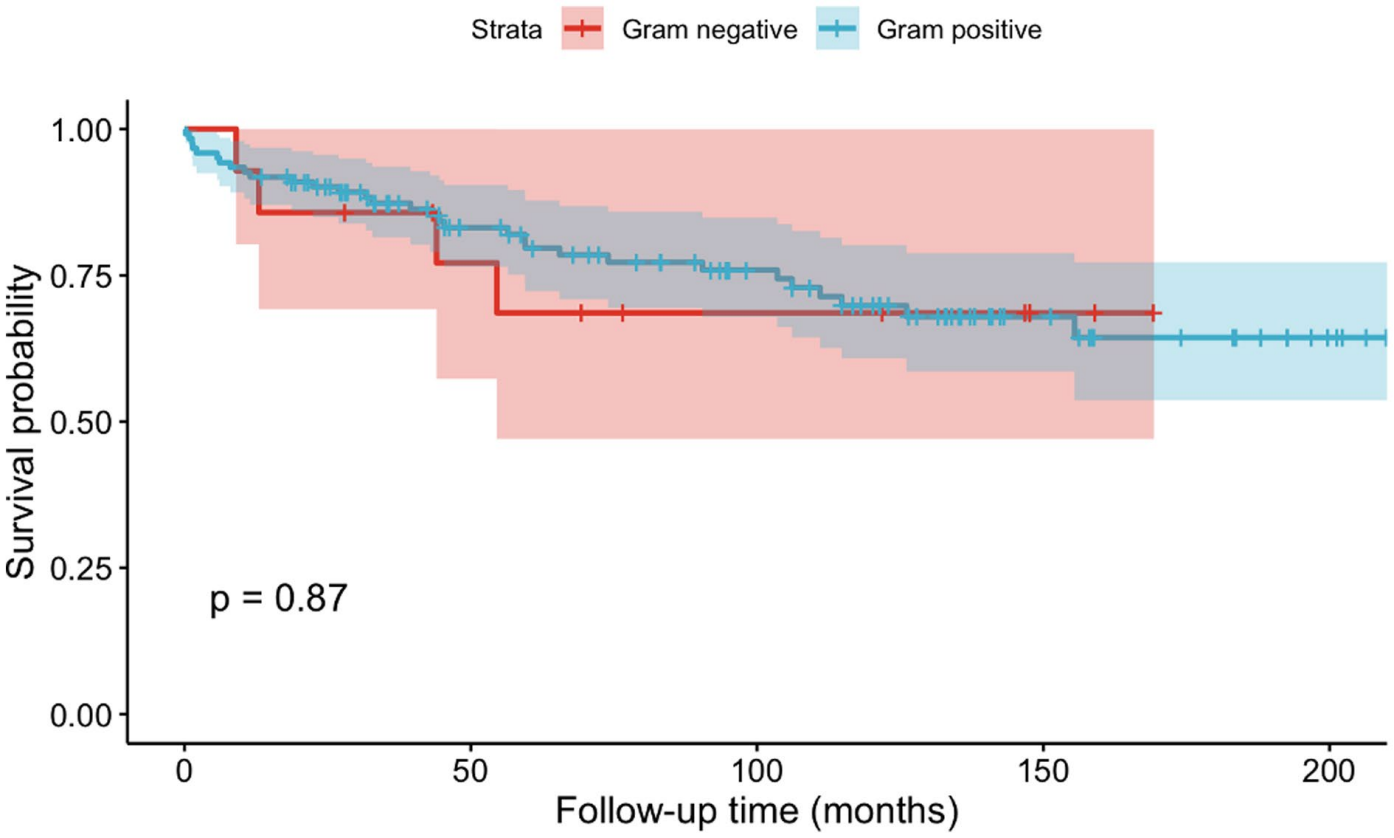
Kaplan–Meier curves depicting mortality based on gram positive or gram negative causative agents.

No significant differences in survival were detected between patients with gram-positive and gram-negative pathogens (*p* = 0.87, cf. Figure 3) regarding mortality. Five patients, including four knee cases and one shoulder case, died within 90 days of admission, which results in a 90-day mortality of 3.68%. Five patients, 4 knees and 1 shoulder case, died within the first 90 days after admission, resulting in an overall 90-day mortality of 3.68%. Three of the cases had an infection with *MSSA*, while two of them had *Streptococcus* spp. as causative agent.

Regarding age groups, we found a decrease in *MSSA* infections and an increase in *Streptococcus* spp. over the age of 65, however this was not statistically significant (*p* = 0.16).

### Susceptibility results

Among all tested antibiotics, the only significant differences over the three time intervals were found for penicillin with a peak of resistances at 37.50% in the from 2007 to 2012 (*p* = 0.004). Further, clindamycin showed resistance rates of 10.71% from 2007–2012, which peaked in the period from 2013 to 2018 at 40.82% and declined again to 32.08% from 2019 to 2024 (*p* = 0.014). No differences in resistance patterns were identified for all other antibiotics over the studied period (cf. Table 5).

**Table 5:** Results of sensitivity testing’s for all three time intervals in absolute numbers and percentages.

Antibiotics	2007–2012 (*n* = 31)		2013–2018 (*n* = 58)		2019–2024 (*n* = 47)		*p*-value
	*S*	*R*	*S*	*R*	*S*	*R*	
Penicillin G	15 (62.5%)	9 (37.50%)	9 (100%)	0 (0.00%)	13 (100%)	0 (0.00%)	**0.004**
Cefotaxim	2 (100%)	0 (0.00%)	3 (100%)	0 (0.00%)	4 (100%)	0 (0.00%)	1.00
Clindamycin	25 (89.29%)	3 (10.71%)	29 (59.18%)	20 (40.82%)	30 (76.92%)	9 (32.08%)	**0.014**
Vancomycin	21 (100%)	0 (0.00%)	11 (100%)	0 (0.00%)	10 (100%)	0 (0.00%)	1.00
Ciprofloxacin	7 (87.50%)	1 (12.50%)	40 (86.96%)	6 (13.04%)	5 (100%)	0 (0.00%)	1.00
Gentamicin	22 (95.65%)	1 (4.35%)	42 (93.33%)	3 (6.67%)	28 (96.55%)	1 (3.45%)	1.00
Erythromycin	23 (82.14%)	5 (17.86%)	27 (61.36%)	17 (38.64%)	26 (78.79%)	7 (21.21%)	0.11
Doxycyclin	23 (100%)	0 (0.00%)	42 (95.45%)	2 (4.55%)	31 (93.94%)	2 (6.06%)	0.68
Tigecyclin	17 (100%)	0 (0.00%)	40 (100%)	0 (0.00%)	26 (100%)	0 (0.00%)	1.00
Fosfomycin	22 (100%)	0 (0.00%)	35 (89.74%)	4 (10.26%)	20 (95.24%)	1 (4.76%)	0.30
Fusidic acid	21 (95.45%)	1 (4.55%)	39 (100%)	0 (0.00%)	26 (100%)	0 (0.00%)	0.25
Linezolid	21 (100%)	0 (0.00%)	45 (100%)	0 (0.00%)	29 (100%)	0 (0.00%)	1.00
Mupirocin	12 (100%)	0 (0.00%)	28 (100%)	0 (0.00%)	24 (100%)	0 (0.00%)	1.00
Rifampicin	19 (100%)	0 (0.00%)	38 (97.44%)	1 (2.56%)	26 (100%)	0 (0.00%)	1.00
Trimethoprim	5 (100%)	0 (0.00%)	41 (97.62%)	1 (2.38%)	16 (94.12%)	1 (5.88%)	0.57
Ampicillin	1 (100%)	0 (0.00%)	2 (66.67%)	1 (33.33%)	3 (75.00%)	1 (25.00%)	1.00
Amoxicillin/ Clavulansäure	1 (100%)	0 (0.00%)	4 (100%)	0 (0.00%)	2 (100%)	0 (0.00%)	1.00
Piperacillin/ Tazobactam	1 (100%)	0 (0.00%)	4 (100%)	0 (0.00%)	5 (100%)	0 (0.00%)	1.00
Cefepim	1 (100%)	0 (0.00%)	6 (100%)	0 (0.00%)	4 (80.00%)	1 (20.00%)	0.50
Amikacin	1 (100%)	0 (0.00%)	6 (100%)	0 (0.00%)	5 (100%)	0 (0.00%)	1.00
Teicoplanin	14 (100%)	0 (0.00%)	5 (100%)	0 (0.00%)	1 (100%)	0 (0.00%)	1.00
Moxifloxacin	1 (100%)	0 (0.00%)	4 (100%)	0 (0.00%)	3 (100%)	0 (0.00%)	1.00
Ceftazidim	1 (100%)	0 (0.00%)	3 (100%)	0 (0.00%)	4 (100%)	0 (0.00%)	1.00
Imipenem	1 (100%)	0 (0.00%)	2 (100%)	0 (0.00%)	2 (66.67%)	1 (33.33%)	1.00

Values in absolute numbers (*n*) and percentages (%), “*S*” meaning susceptible, “*R*” meaning resistance given.

## Discussion

In this 18-year retrospective study, we found that gram-positive cocci remained the primary pathogens causing native joint septic arthritis of the knee and shoulder, with *S. aureus* being by far the most prevalent causative agent in about half of all cases. Our findings are consistent with previous studies, showing *S. aureus* rates of 45% for the knee joints and 47% for the shoulders in central Europe (19), as well as values of about 66% for knees (20) and 39% (21) for shoulders in northern America.

Regarding sensibility, only six cases of *MRSA*, equalling 8.6% of all *S. aureus* infections and 4.4% of all infections, were identified in our cohort, five of which occurred between 2013 and 2018. This temporal pattern aligns with previous literature on *S. aureus* septic arthritis, as Weiss et al. reported a decline in *MRSA* rates among pediatric patients from 45% in 2009 to 18.2% in 2016, compared to *MSSA* infections (22). Additionally, global surveillance data over the last twenty years showed similar patterns in the proportion of various types of *MRSA* infections, with a steady decline over the last decade (23), which was also noted in the Austrian national report on antibiotic susceptibility from 2022 (24). However, global differences in *MRSA* rates must be taken into consideration in the process of creating guidelines for empirical antibiotic therapy (23), as rates on a spectrum from 8.2% in Israel (25) to 23% in the USA (20) were reported, and higher *MRSA* rates in the USA were at least partly linked to the ongoing opioid epidemic (20).

Based on our data, the liberal use of vancomycin, linezolid or daptomycin cannot be supported in central Europe. If however, an *MRSA* infection is given, we found no resistances against one of these antibiotics, which is in line with almost identical results to the overall Austrian data for microbial resistances, reporting *MRSA* rates of 3.9% in 2022 and no relevant resistances against vancomycin (24). Therefore, it should still be considered first line i.v. therapy in case of *MRSA* (25, 26). Overall, *S. aureus* has evolved through successive waves of dominant clones over the years, each with distinct antimicrobial susceptibility patterns, underscoring the need for ongoing surveillance in the future (27).

In terms of antibiotic susceptibility, penicillin G showed a significant rate of resistance in the earliest study period (2007–2012), with 37.5% of isolates affected, which was no longer observed in the subsequent decade. Similar trends were observed by the SENTRY antimicrobial surveillance program for *S. aureus*, with susceptibility rates for penicillin rising between 1997 and 2016 from 14 to 26% (23). As for clindamycin, resistance rates differed significantly over time, with highest rates found from 2013 to 2018 at 40.82%, remaining persistently high at 32.08% from 2019 to 2024.

Despite being well tolerated and having excellent tissue penetration properties (28), the liberal use of clindamycin for patients with suspected penicillin allergy and staphylococcal, streptococcal, and gram-positive anaerobic bacterial infections might have contributed to rising resistance rates. This further underscores the importance of tests such as the erythromycin-clindamycin D-zone test to assess potential of developing clindamycin resistance (29).

Given the low true rate of clinically significant penicillin hypersensitivity and the well-documented risks of broad-spectrum antibiotics, the liberal use of clindamycin in patients with suspected penicillin allergy in orthopedic surgery should be reconsidered to align with antimicrobial stewardship principles (30). True penicillin hypersensitivity is rare and affects fewer than 5% of patients who report an adverse reaction to penicillin in the past. Importantly, cross-reactivity with cephalosporins is not primarily caused by the *β*-lactam ring itself but due to structural similarities in the R1 side chains (31). When side chains differ, the risk of cross-reactivity is very low, whereas identical or closely related side chains (for example between ampicillin and cephalexin) can confer a higher risk (32), but even in those with proven IgE- or T-cell-mediated penicillin allergy, cross reactivity with cephalosporins remains as low as 16.45% (33). Therefore, the use of easily applicable assessment tools such as the PEN-FAST test is highly encouraged to guide antibiotic prescriptions in septic arthritis, even in emergency settings (34).

No significant changes over time were observed for any of the other antibiotics. Further, no significant differences in pathogen distributions between patients suffering from septic arthritis after intraarticular injections compared to those with septic arthritis from other causes were identified. Incidences of other coagulase negative staphylococci showed a constant decline over time, with rates as low as 2.13% from 2019 to 2024. Gram-negative bacteria only account for around 10–20% of cases in literature (10), most often appearing in patients with a history of intravenous drug abuse or immunocompromisation (35), and accordingly showed almost constant rates of 10% over the observed period of 18 years in this study. Moreover, only a single fungal infection was detected in our cohort. Empirical therapy at our institution therefore currently consists of a first-generation cephalosporin or an anti-staphylococcal penicillin in line with recent recommendations by the European Bone and Joint Infection Society, and is initiated right after appropriate samples were obtained (9). Additional *MRSA*-specific coverage is not routinely used, as the local prevalence of *MRSA* is well below 15% (9). Overall, narrow spectrum antibiotics, reduction of vancomycin and multi-drug use, and treatment durations as short as possible are chosen to minimize resistance development and *Clostridioides difficile* infections according to antibiotic stewardship guidelines (36). Nevertheless, a treatment duration of at least 4 weeks is recommended, as shorter durations have been associated with a higher risk of failure in culture-positive septic arthritis (37).

Our cohort showed a 90-day mortality rate of 3.68%, which is consistent with recent findings for septic arthritis of the knee reported by Choi et al. in Korea with 2.7% (38). In contrast, Huang et al. reported higher mortality rates of up to 8.6% when assessing infections of the knee, shoulder, hip, and multiple joints, and their survival analysis demonstrated significant differences in mortality depending on the joint involved (39). These findings highlight the importance of considering the affected joint when evaluating mortality risk.

Further, of the five patients (3.68%) who died within 90 days of admission, three had *S. aureus* infections and two had *Streptococcus ssp.* infections. This distribution is consistent with previous reports identifying *S. aureus* as the most common pathogen in septic arthritis and one that carries a higher risk of adverse outcomes and mortality (40). Although *Streptococcus* spp. are considered less virulent, they also accounted for a considerable proportion of fatal cases in our series, which aligns with prior data suggesting intermediate mortality risk compared to *S. aureus*, especially in older or immunocompromised patients (2). Additionally, our findings show an age-related distribution of pathogens, with *S. aureus* infections more common in patients aged 18–64 years and *Streptococcus* spp. predominating in those over 65 years, however, the trend was not statistically significant. This pattern is consistent with previous reports, which demonstrated a decline in *S. aureus* prevalence with increasing age and a higher frequency of streptococcal infections in older populations (20). Thus, our findings underscore the previously demonstrated pathogen-specific and age-dependent impact on diagnosis and prognosis.

This study has several limitations. Its retrospective design limits control over data quality. However, cases were defined using only aspiration results with positive microbiology and a high clinical suspicion of septic arthritis was given, reducing the risk of misclassification to a minimum. As a single-center analysis, generalisability is limited, but our hospital serves as the major referral center in Austria. Therefore, our data can be thought of as representative for the local pathogen distribution, but further studies and multinational surveillance measures are needed. Detailed antimicrobial treatment data were not collected, but cases are routinely discussed with infectious disease specialists from our hospital. Lastly, most cases were community-acquired native joint infections, which may explain the low prevalence of resistant pathogens.

Overall, a thorough understanding of geographical differences in microbes, including temporal trends, is essential to guide empirical therapy and support appropriate antimicrobial use. Septic arthritis of large joints such as the knee and shoulder represents a clear example in which comprehensive antimicrobial and diagnostic stewardship need to be fully integrated to maintain antibiotic effectiveness and ensure optimal outcomes.

## Data Availability

The original contributions presented in the study are included in the article/supplementary material, further inquiries can be directed to the corresponding author/s.
